# Structural Fault Detection and Diagnosis for Combine Harvesters: A Critical Review

**DOI:** 10.3390/s25133851

**Published:** 2025-06-20

**Authors:** Haiyang Wang, Liyun Lao, Honglei Zhang, Zhong Tang, Pengfei Qian, Qi He

**Affiliations:** 1 College of Agricultural Engineering, Jiangsu University, Zhenjiang 212013, China; 2222316044@stmail.ujs.edu.cn (H.W.); 2112416030@stmail.ujs.edu.cn (H.Z.); 2 Faculty of Engineering and Applied Sciences, Cranfield University, Cranfield MK43 0AL, UK; 3 Key Laboratory of Modern Agricultural Equipment and Technology, Ministry of Education, Jiangsu University, Zhenjiang 212013, China; 4 School of Mechanical Engineering, Jiangsu University, Zhenjiang 212013, China; pengfeiqian@ujs.edu.cn (P.Q.); 2222452011@stmail.ujs.edu.cn (Q.H.)

**Keywords:** combine harvester, fault detection, fault diagnosis, data-driven methods, machine learning, signal processing

## Abstract

Combine harvesters, as essential equipment in agricultural engineering, frequently experience structural faults due to their complex structure and harsh working conditions, which severely affect their reliability and operational efficiency, leading to significant downtime and reduced agricultural productivity during critical harvesting periods. Therefore, developing accurate and timely Fault Detection and Diagnosis (FDD) techniques is crucial for ensuring food security. This paper provides a systematic and critical review and analysis of the latest advancements in research on data-driven FDD methods for structural faults in combine harvesters. First, it outlines the typical structural sections of combine harvesters and their common structural fault types. Subsequently, it details the core steps of data-driven methods, including the acquisition of operational data from various sensors (e.g., vibration, acoustic, strain), signal preprocessing methods, signal processing and feature extraction techniques covering time-domain, frequency-domain, time–frequency domain combination, and modal analysis among others, and the use of machine learning and artificial intelligence models for fault pattern learning and diagnosis. Furthermore, it explores the required system and technical support for implementing such data-driven FDD methods, such as the applications of on-board diagnostic units, remote monitoring platforms, and simulation modeling. It provides an in-depth analysis of the key challenges currently encountered in this field, including difficulties in data acquisition, signal complexity, and insufficient model robustness, and consequently proposes future research directions, aiming to provide insights for the development of intelligent maintenance and efficient and reliable operation of combine harvesters and other complex agricultural machinery.

## 1. Introduction

Grain crops are staple food globally and important industrial raw materials [1,2,3], and harvesting operations are a critical link in the grain production process [4,5]. Grain combine harvesters (referred to as combine harvesters), as core equipment in modern agricultural production systems, integrate multiple complex functions, significantly reducing labor input and improving crop harvesting efficiency and yield. They must operate for extended periods in vast fields, adapting to diverse environments. Their efficient and reliable operation is a cornerstone for ensuring food security and sustainable agricultural development. Crucially, harvesting is a highly time-sensitive operation, confined to a narrow window when crops reach optimal maturity and moisture content. Any unexpected downtime during this critical period can lead to significant economic consequences. These extend beyond direct repair costs to include yield loss due to untimely harvesting, degradation of crop quality, and increased subsequent operational costs. Therefore, the economic value of preventing even a single day of downtime during peak season can be substantial. However, the complex structure, high operational loads, and severe working environments of combine harvesters pose significant challenges [6]. Structural faults are one of the primary factors influencing their performance and reliability.

During field operations, agricultural machinery is continuously exposed to environments such as dust, moisture, vibration, impact, and variable loads [7,8,9,10,11,12,13]. These conditions cause continuous and irregular stress and damage to structural components [14]. Common structural faults include wear and fatigue fracture of transmission components [15], loosening of critical connecting parts [16], unbalance, deformation, and fatigue of working parts [17], as well as blockages and damage to related components caused by abnormal material flow [18,19,20,21,22].

**Figure 1:**
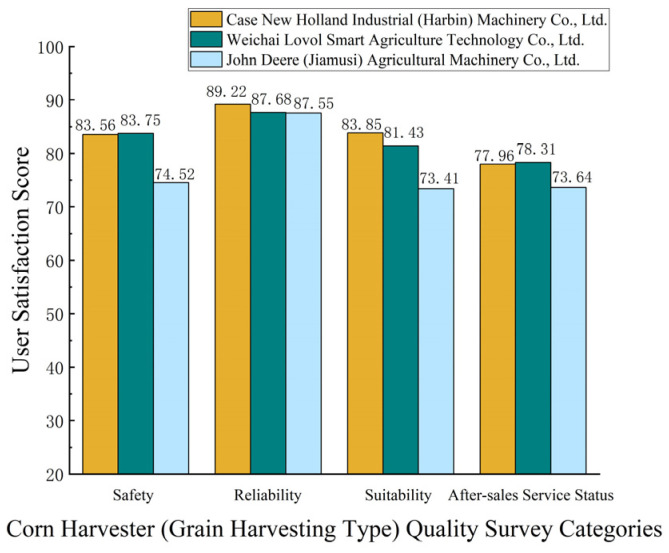
User satisfaction evaluation results from the 2023 corn combine harvester (grain type) quality survey.

**Figure 2:**
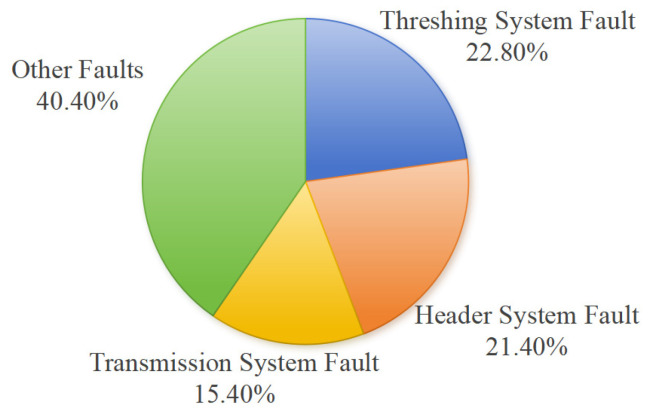
Fault types and their proportions in the surveyed corn combine harvesters.

In 2023, the Department of Agricultural Mechanization Management of the Ministry of Agriculture and Rural Affairs of China conducted a quality survey on some in-service corn combine harvesters (grain type) from different companies. The survey adopted a user satisfaction evaluation method, where users individually evaluated the satisfaction levels for four aspects of the corn combine harvesters they used: safety, reliability, applicability, and after-sales service. The results of the satisfaction evaluation are shown in Figure 1. Simultaneously, statistics showed that during operation, 22.98% of the surveyed machines experienced a total of 298 faults. Among these, faults in the threshing body, header system, and transmission system accounted for a relatively high proportion of the total number of faults, as shown in Figure 2. These faults not only lead to performance degradation, increased losses, and increased energy consumption, but also pose significant risks to the health and safety of operators and on-site personnel [23]. Therefore, developing accurate and timely Fault Detection and Diagnosis techniques is crucial for minimizing operational interruptions, enhancing overall system reliability, and ultimately contributing to global food security and promoting agricultural sustainability [24,25].

The practical value of fault diagnosis is further underscored by real-world maintenance experiences. For instance, according to a report on agricultural machinery maintenance cases by the China Agricultural Mechanization Association, older combine harvesters frequently exhibit higher grain loss rates and incur substantial repair costs, posing significant challenges for farmers. To address this, a novel approach, utilizing ANSYS software for finite element analysis, has been successfully applied to optimize the threshing unit of older combine harvesters. By simulating component wear and optimizing adjustments, this ANSYS-based strategy significantly reduced threshing unit repair frequency by 50% and maintenance costs by 80%, substantially decreasing grain loss and extending machine lifespan, thereby demonstrating tangible improvements in operational efficiency and economic benefits.

Fault Detection and Diagnosis (FDD) for combine harvester faults is central to improving harvester reliability [26,27]. Traditional methods rely on manual inspection and empirical methods, which have limited efficiency and accuracy. The structural complexity of combine harvesters, variable working conditions, scarcity of fault samples, non-stationarity of signals, and strong noise pose significant challenges for FDD.

In recent years, with the continuous development of sensor technology, data science, and computing capabilities [28,29,30,31,32,33,34], fault diagnosis is progressing towards greater intelligence [35]. Data-driven methods offer opportunities for addressing FDD challenges [36,37]. Data-driven methods are capable of analyzing data and automatically learning fault patterns without requiring precise physical models [38]. Signal processing techniques are employed to extract features from complex signals [39]. Subsequently, machine learning and artificial intelligence algorithms enable automatic identification and diagnosis, improving efficiency and accuracy. Furthermore, the development of remote monitoring and on-board diagnostic platforms provides technical support for the application of these methods.

Considering the significance of FDD for structural faults in combine harvesters and the advancements in data-driven methods, this review aims to provide a systematic review and analysis of the progress of data-driven methods in this domain, with potential applicability to other types of agricultural machinery facing similar challenges. Particular attention is paid to signal analysis, feature extraction, and the application of machine learning and artificial intelligence models. The structure of this paper is organized as follows: Section 2 will review and summarize the typical structural components of combine harvesters and their related common structural fault types; Section 3 will delve into the specific methods of data-driven structural fault detection and diagnosis, including data acquisition and preprocessing, signal processing and feature extraction, and the application of machine learning and artificial intelligence models; Section 4 will discuss the main challenges currently faced in this field; Section 5 will outline future research directions; and Section 6 provides a summary of the entire paper.

## 2. Typical Structure and Fault Types of Combine Harvesters

### 2.1. Overview of the Structural Composition of Combine Harvesters

**Figure 3:**
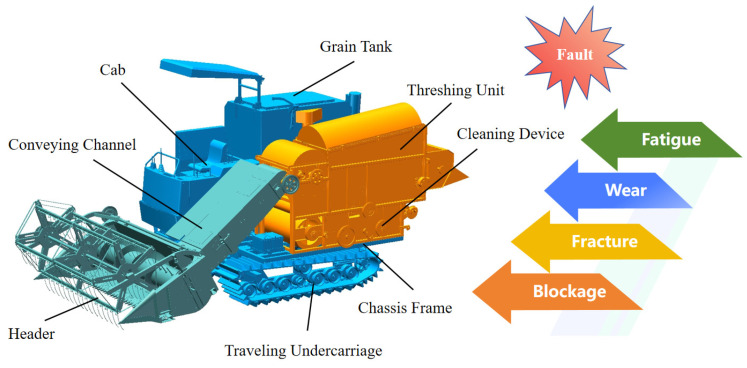
Typical structure of a combine harvester.

A combine harvester is a comprehensive platform that highly integrates multiple complex mechanical, hydraulic, electrical, and electronic systems. Its main workflow covers steps such as crop cutting, feeding, threshing, separation, cleaning, and grain collection [40,41,42,43]. To achieve these functions, a combine harvester is composed of multiple key structural modules, including the header located at the front of the machine for cutting crops [44]; a feeding section that evenly feeds crops into the interior; a threshing and separation section that achieves separation of grain from straw [45,46,47]; a cleaning section for removing impurities and obtaining clean grain [48,49,50]; a grain tank for temporary storage of grain; and an unloading auger for unloading grain into transport vehicles. Furthermore, the power driving these working modules primarily comes from the engine and is transmitted through complex transmission systems (including gearboxes, bearings, belts, chains, etc.). The hydraulic system is responsible for controlling actions such as lifting and lowering the header, rotating the unloading auger, and steering. The entire machine moves and is supported in the field by the chassis frame and traveling undercarriage. These modules contain a large number of structural components, whose interactions and complexity determine the diversity of structural faults. A typical combine harvester structure is shown in Figure 3.

### 2.2. Typical Fault Types of Key Structural Components

**Figure 4:**
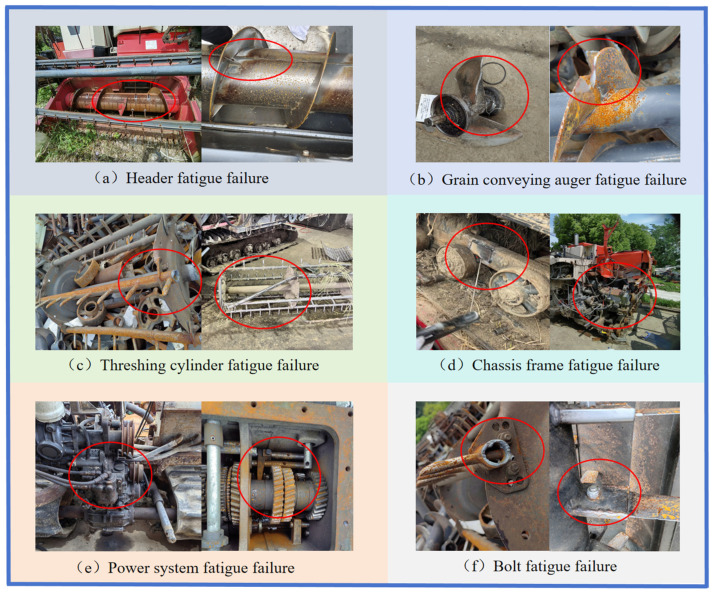
Examples of fatigue failure in various components of a combine harvester. The red circles indicate the specific locations of fatigue failure noted in each figure.

Components within the structural modules of combine harvesters operating for long periods under high loads and harsh field environments are highly prone to various types of structural faults [51,52]. These faults directly impact the function of the components and the overall performance of the machine. These fault types are diverse, including, for example, fatigue, wear, fracture, and blockage. Among these, fatigue is one of the common modes leading to structural component failure. Figure 4 shows examples of fatigue failure in some key components of combine harvesters.

Analysis of typical fault types of key structural components of combine harvesters includes the following components:

(1) Header and Feeding Section: The header is the component that first contacts the crops [53], and its cutter blades are prone to wear and fracture. The feeding auger and chain conveyor within the feeding section are prone to blockages when handling wet or tangled materials, leading to component overload and accelerated wear [54,55]. For instance, Li et al. [56] investigated the issues of material backflow and blockage in the feeding auger and chain conveyor of a rapeseed combine harvester header when handling rapeseed straw. Through optimized design, they improved conveying efficiency and seed shedding rate. Su et al. [57] studied the curved spike teeth in a sowing layer residual film recovery machine, finding them susceptible to wear and fracture deformation in soil environments containing abrasive particles, and analyzed the mechanical properties and wear characteristics of different materials. Yi et al. [58] pointed out the high blockage rate of the threshing drum, which significantly impacts the working efficiency and reliability of combine harvesters.
(2) Threshing Unit: As one of the modules experiencing the highest impact and vibration in combine harvesters, components such as the threshing drum and its bearings, and the separation drum are common sources of faults [59,60,61,62]. For example, Bhandari et al. [63] studied the vibration analysis of threshing drum bearings, applying it to the mechanical fault diagnosis of the threshing unit of a combine harvester. Jotautiene et al. [64] also investigated diagnostic methods for threshing drum rolling bearings, highlighting the challenges of detection in locations where sensors are difficult to install. Frequent faults in threshing drum bearings, such as wear, pitting, and spalling, are key reasons for machine performance degradation and downtime. Long-term wear or deformation of the drum can lead to dynamic imbalance issues, causing severe machine vibration. Cheng et al. [65] studied the effect of drum screen parameters on corn cob blockage patterns in a corn grain combine harvester, showing that optimizing motion parameters can reduce blockages. Material flow blockages can also affect other components, for example, leading to overload of straw augers or unloading augers.
(3) Cleaning Section: The cleaning sieve is a core component, and its wear resistance and anti-blockage performance are crucial [66,67,68,69,70]. Ma et al. [17] tested and analyzed the durability of the cleaning sieve. Cheng et al. [38] studied the design and experiments of a coated screen for rice combine harvesters, aiming to address sieve adhesion and blockage issues. They also investigated the blockage patterns and peeling tests of the sieve in corn grain combine harvesters [71].
(4) Transmission System: This includes gearboxes, bearings, transmission shafts, belts, chains, etc., which are responsible for transmitting engine power to various working modules [72,73,74]. Akinci et al. [75] analyzed the failure causes of transmission gears in rotary tillers, finding wear and plastic deformation to be the main fault types, with design and material defects as the root causes. Li et al. [76] analyzed the fatigue failure issue of the Hydrostatic Transmission (HST) differential gearbox in crawler-type harvesters and proposed a fault diagnosis method based on order analysis. Xue et al. [77] studied fault diagnosis methods for the wet clutch control system in tractors. Relevant faults may be related to structural issues such as seal damage or oil circuit blockage. Yan et al. [78] investigated the load spectrum of the tractor Power Take-Off (PTO) shaft during rotary tillage operations and used it for evaluating fatigue damage.
(5) Hydraulic System: The hydraulic system controls functions such as lifting and lowering of the machine, steering, and unloading. Its faults are often related to structural or performance degradation issues such as valve blockage caused by oil contamination, and leakage caused by component wear [79]. Rogovskii et al. [80] pointed out that failures of individual components in the hydraulic system can affect the function of the entire subsystem and explored methods for diagnosing the technical state of the hydraulic system based on external features. Chen et al. [81] proposed a new hydraulic system fault diagnosis method that utilizes vibration signals obtained from a hydraulic motor for analysis. Xiong et al. [82] investigated the issue of slight internal leakage in check valves and proposed an algorithm based on multi-source, multi-domain, multi-scale feature extraction and machine learning. Experimental results indicated that this algorithm can effectively detect leakage, with a leakage mode recognition rate exceeding 90%. Wang et al. [83] analyzed the impact of hydraulic system faults (e.g., seal ring damage, oil circuit blockage) on the shifting quality of CVT tractors.
(6) Chassis Frame and Connecting Parts: The chassis frame, serving as the supporting structure of the machine, must withstand the entire weight of the machine and field impacts, and is prone to structural deformation or fatigue damage. For example, Kim et al. [84] emphasized the structural safety of front loaders for agricultural machinery. Mattetti et al. [85] evaluated the fatigue damage of agricultural tractor axle housings, finding that the most damaging events during field operation occur during headland turns. Wen et al. [86] designed accelerated structural durability test methods for tractors, aiming to address the issues of unreasonable test design and unsystematic analysis. Xu et al. [16] specifically studied detection and diagnosis methods for loosened bolts in vibrating screens, proposing a method based on time–frequency analysis of vibration signals and evaluated the effectiveness of the proposed method by training and testing classification decision models.

Based on the analysis of typical faults and their occurrences across different structural components, and informed by industry statistics such as the 2023 quality survey (as shown in Figure 2) and the broader literature [53,54,55,56,57,58,59,60,61,62,63,64,65,66,67,68,69,70,71,72,73,74,75,76,77,78,79,80,81,82,83,84,85,86], a likelihood matrix is compiled in Table 1, illustrating the estimated likelihood (H—high, M—medium, L—low) of key fault types occurring in various sections of the combine harvester.

### 2.3. Progressive and Abrupt Nature of Structural Faults

Structural faults in combine harvesters exhibit diversity during their development. Some faults, such as component wear, fatigue crack propagation, or exacerbated unbalance [7,87], are progressive processes that accumulate slowly. Their early signals are often weak, requiring advanced detection techniques for timely capture [88]. For instance, Xie et al. [89] investigated the early fault diagnosis of rolling bearings in agricultural machinery, highlighting the challenges posed by small sample size and imbalanced data for early fault identification. In contrast, other faults, such as sudden component fracture or severe blockage, may manifest as abrupt events. Understanding the characteristics of fault occurrence and development is crucial for selecting appropriate detection and diagnosis methods.

## 3. Data-Driven Methods for Structural Fault Detection and Diagnosis

**Figure 5:**
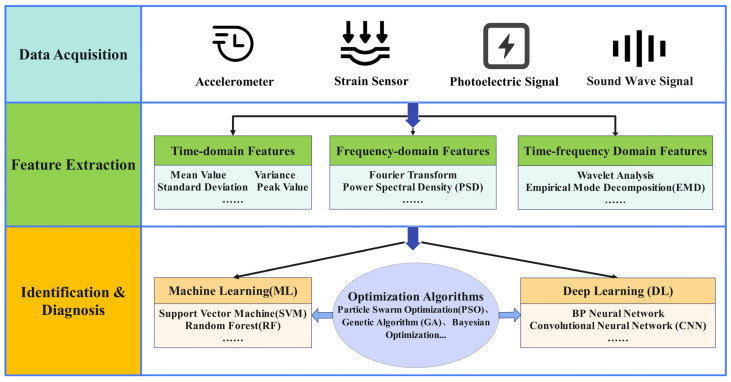
Flowchart of structural fault detection and diagnosis.

Currently, agricultural machinery, including but not limited to combine harvesters, are evolving towards large-scale, intelligent, efficient, and multi-functional development [90,91,92,93,94,95,96,97,98]. Data-driven structural fault detection and diagnosis methods for combine harvesters fully leverage various signals collected from machine operation. Through signal processing and machine learning techniques, they enable the evaluation of structural health status and the identification of fault types. This process typically includes data acquisition and preprocessing, signal processing and feature extraction, and the application of machine learning and artificial intelligence models. A flowchart of structural fault detection and diagnosis is shown in Figure 5.

### 3.1. Data Acquisition and Preprocessing

Data serve as the basis for data-driven FDD. In complex and variable agricultural machinery operating environments, effectively and reliably collecting signals that reflect the structural status is the primary challenge.

(1) Signal Types and Acquisition Equipment

**Figure 6:**
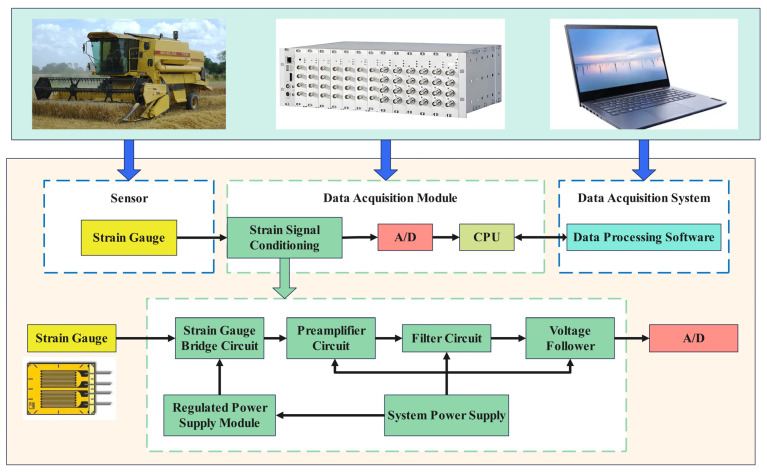
Schematic diagram of the load acquisition system.

Vibration signals are the most commonly used signal type in FDD for structural faults of combine harvesters, particularly for monitoring the health status of rotating parts (e.g., bearings, gears, drums) [99,100]. For example, Chen et al. [81] used vibration signals for fault diagnosis of hydraulic systems. Accelerometers are typically used for measuring vibration signals [101]. Acoustic signals (acquired by microphones) can be used for monitoring abnormal sounds, and some studies have explored using smartphone microphones to acquire acoustic data for agricultural machinery health diagnosis [102]. Force sensors or strain gauges can be used to measure the force or strain on structural components, directly reflecting the load and fatigue status [103]. By placing strain gauges on key parts of the combine harvester, actual load or response signals are acquired under typical working conditions. The load acquisition system includes sensors, data acquisition modules, and data acquisition software, where the data acquisition software is installed on a laptop. A schematic diagram of the load acquisition system is shown in Figure 6.

Rotational speed sensors are used to monitor the rotational speed of components and are important parameters for diagnosing transmission faults and blockages. Fault detection in hydraulic systems typically relies on pressure sensors, flow meters, or actuator position sensors [104]. On-board CAN bus data can provide abundant information about the machine’s operating status. Furthermore, image recognition technology is also being explored for detecting surface defects of agricultural machinery components [105]. Signal acquisition equipment is usually connected to a data acquisition system. Studies have shown that the placement of sensors affects signal quality and diagnostic effectiveness [106], and optimizing sensor layout can improve diagnostic accuracy [107]. Table 2 summarizes and compares the advantages, disadvantages, and applicability to field conditions of common signal types.

(2) Data Preprocessing

The collected raw signals often contain environmental noise, sensor errors, and interference caused by varying working conditions. Preprocessing is necessary to improve data quality and the effectiveness of subsequent analysis. Common preprocessing techniques include filtering, which is used to remove noise within specific frequency ranges. Signal decomposition methods, such as Empirical Mode Decomposition (EMD) or Variational Mode Decomposition (VMD) and their variants, can decompose the raw signal into different modal components, which is helpful for separating noise and fault-related components. Denoising Autoencoder (DAE) is a deep learning method that removes noise by learning a low-dimensional representation of the data [108]. Singular Value Decomposition (SVD) has also been applied to the denoising of vibration signals, aiming to address complex noise interference issues. Some methods attempt to combine optimization algorithms to adaptively adjust denoising parameters, thereby achieving better denoising results [109]. Furthermore, for multi-source signals, synchronous processing is also required, for example, methods based on maximum cross-correlation [110].

Data acquisition serves as the foundation for data-driven FDD, with signal quality and diversity directly impacting the accuracy of subsequent analysis. Current research has explored various sensor types and signal processing techniques to address the complex, noisy, and non-stationary signal characteristics encountered in combine harvesters. Nevertheless, reliable and long-term signal acquisition in challenging field environments remains a significant challenge, particularly concerning the capture of early weak signals and abrupt faults. Future research endeavors should prioritize the optimized deployment of sensor networks, enhance the reliability of wireless data transmission, and develop customized signal acquisition strategies tailored to specific combine harvester components and fault types. Furthermore, exploring more advanced adaptive preprocessing techniques is essential to maximize the retention of crucial fault-related information while effectively suppressing noise and interference.

### 3.2. Signal Processing and Feature Extraction

Extracting features from preprocessed signals that can effectively reflect the structural fault status is a core step in data-driven FDD. Researchers have explored various signal processing and feature extraction techniques.

(1) Time-Domain Features

Features are quantified by calculating the statistical properties of the signal in the time domain. Common time-domain features include mean, variance, standard deviation, root mean square (RMS), peak value, kurtosis, skewness, peak-to-peak value, clearance factor, shape factor, impulse factor, etc. These features can reflect information such as signal energy, fluctuation, and impulsiveness. For example, Abad et al. [39] extracted descriptive statistical parameters from the acoustic signals of the differential as fault features. Wen et al. [111] calculated statistical parameters, such as mean, RMS, and kurtosis, from the load signals of the tractor front axle and used them for evaluating fatigue damage, finding that these parameters were consistent with the variation trend of the original load signals.

(2) Frequency-Domain Features

Signals are transformed into the frequency domain through Fourier Transform (FFT) or Power Spectral Density (PSD) analysis to extract frequency components that reflect periodic impacts and vibrations. Frequency-domain features typically include amplitude, energy, or power spectral density values at specific frequencies, particularly components related to component rotational frequency, gear mesh frequency, or bearing fault characteristic frequencies [112]. For instance, Bhandari et al. [63] used FFT analysis of vibration signals from threshing drum bearings to identify fault frequencies. Feijoo et al. [113] employed composite spectrum analysis for unbalance faults in multiple rotating components of a combine harvester, finding that the composite spectrum can effectively identify rotation-related frequency peaks and reduce analytical complexity.

(3) Time–Frequency Features

Signals generated during agricultural machinery operation are often non-stationary. Time–frequency analysis methods can simultaneously capture changes in the signal over time and frequency, making them suitable for analyzing transient features and signals under varying working conditions. Wavelet Transform (WT) can provide multi-resolution analysis, which is suitable for detecting transient features. Hilbert–Huang Transform (HHT) and its improved versions can provide adaptive time–frequency decomposition of signals, suitable for non-linear and non-stationary signal analysis [114]. Short-Time Fourier Transform (STFT) is also a commonly used time–frequency analysis technique [115]. Through time–frequency analysis, time–frequency distribution maps of the signal can be obtained, from which time–frequency features reflecting faults can be extracted, for example, for identifying threshing drum blockage [116].

(4) Modal Analysis and Decomposition

Modal analysis decomposes signals into modal components with specific physical meaning, which is helpful for separating signals generated from different fault sources or reducing noise. Variational Mode Decomposition (VMD) is an advanced adaptive signal decomposition method that has been used to analyze weakly damped signals in tractor engine vibration and noisy environments [117]. Parameter-adaptive VMD (PAVMD) has been utilized to decompose the non-stationary vibration signals of the threshing drum for unbalance detection [118]. Empirical Mode Decomposition (EMD) and its improved versions (e.g., EEMD) have also been applied to the decomposition of vibration or pressure signals to extract fault features. Through modal decomposition, the time series or time–frequency spectra of each modal component can be obtained, and their features can be further extracted.

(5) Other Advanced Features

This includes measures of signal complexity, such as information entropy or sample entropy, which can reflect the regular variations in a signal and are used for detecting anomalies. Multi-scale Reverse Dispersion Entropy (MRDE) and Optimized Multi-scale Reverse Dispersion Entropy (OMRDE) have been used to extract vibration signal features for combine harvester assembly faults [119]. Sparse representation extracts atoms or dictionary elements from signals that best represent their characteristics, used for fault detection [120]. Physics-based model-driven methods have also been utilized to guide the selection of wavelet packet decomposition coefficients, allowing them to better reflect gear damage characteristics. Shape feature extraction focuses on capturing specific patterns in time series data and is applicable to identifying operational fault modes [121].

Signal processing and feature extraction are core technical steps in data-driven FDD methods. Existing research has explored a wealth of time-domain, frequency-domain, time–frequency, and modal analysis methods capable of extracting features from various sensor signals that reflect the structural fault status. In recent years, emerging adaptive signal processing techniques combined with deep learning-based feature extraction methods have demonstrated advantages in handling the non-stationarity and noise interference characteristic of combine harvester signals. Nevertheless, how to extract features that are most sensitive and physically meaningful for specific structural faults (especially early and compound faults), and how to maintain feature robustness under complex working conditions, remain areas requiring in-depth research.

### 3.3. Machine Learning and Artificial Intelligence Models

In recent years, machine learning and artificial intelligence technologies have developed rapidly [122,123,124,125,126,127]. After extracting fault features, machine learning and artificial intelligence models can be used to learn fault patterns and perform automatic classification and diagnosis. Various ML/AI models have been applied to FDD of structural faults in combine harvesters and related agricultural machinery [128,129].

(1) Applications of Classic Machine Learning Models

Support Vector Machine (SVM): As a powerful classifier, SVM is widely applied in various structural fault diagnosis tasks due to its good performance on small sample and high-dimensional data. Chen et al. [81] used SVM to diagnose hydraulic system faults. Ni et al. [104] diagnosed electro-hydraulic control system faults based on SVM, achieving a classification accuracy of 90% on test samples. Ruiz-Gonzalez et al. [130] used SVM based on single-point vibration signals to estimate the state of agricultural machinery rotating parts, achieving a cross-validation accuracy of 85% even with a small number of features.

(2) Applications of Deep Learning Models

Neural Networks (NNs): NN and their variants have been used for various fault diagnosis tasks. Chen et al. [131] combined BPNN and DS Evidence Theory to diagnose combine harvester blockages, which improved diagnostic accuracy. Ebrahimi et al. [132] used ANFIS to diagnose starting motor faults in tractors, demonstrating good performance at different rotational speeds. Ma et al. [126] used BPNN to predict the distribution of material on the sieve, which was used for anti-blockage control. Zhou et al. [133] proposed a fault diagnosis model for tractor diesel engines based on a Linear Weight Reduction-Quantum Particle Swarm Optimization-Self-Organizing Map Backpropagation (LWD-QPSO-SOMBP) neural network. Compared to a single BP neural network, the diagnostic accuracy of the LWD-QPSO-SOMBP neural network model was significantly improved. The training accuracy increased from 85.00% to 99.44%, demonstrating excellent diagnostic performance.

Convolutional Neural Network (CNN): CNNs are well-suited for processing grid-like data such as images or time–frequency maps and are widely used for fault diagnosis in bearings, engines, and transmission systems [134]. Shi et al. [135] proposed a diagnosis method combining IHHT and CNN. First, they improved HHT through Multi-population Differential Evolution improved Ensemble Empirical Mode Decomposition (MPDE-EEMD) and a sensitive Intrinsic Mode Function (IMF) screening method to extract time–frequency features of fault signals. Specifically, MPDE-EEMD was used to decompose the raw vibration signals into IMFs, and then a sensitive IMF screening method based on similarity measures was applied to select IMFs that best represented fault characteristics. These selected IMFs were then transformed into time–frequency maps using IHHT. Then, based on the AlexNet network model, they traversed all possible CNN model combinations to construct a CNN model adapted for rolling bearing fault diagnosis. Their proposed CNN architecture consisted of specific convolutional and pooling layers designed to process the IHHT time–frequency maps (images of 256 × 256 pixels). The diagnostic accuracy was evaluated using classification accuracy on both single and compound fault types. Subsequently, IHHT time–frequency maps generated from the training set were input into the CNN for learning, and the model was applied to the test set to output fault identification results. The experimental setup involved two different bearing fault experiments (single faults and compound faults) with data collected under varying loads and speeds. The diagnostic accuracy for single faults was reported as 100%, and for compound faults as 99.74%. Compared to traditional methods, this approach improved diagnostic accuracy. Rajakumar et al. [136] used DCNN based on acoustic signals for agricultural machinery health monitoring and employed LFOA to optimize the network structure for lightweighting. Wang et al. [137] used an improved CNN architecture combined with data fusion and Bayesian optimization to diagnose PMSM short-circuit faults, achieving a validation accuracy of up to 98.2%.

Autoencoder (AE) and Denoising Autoencoder (DAE): These are often used as tools for deep feature extraction. Qiu et al. [138] used SDAE to extract deep non-linear features from combine harvester operational data and combined it with SVM for fault prediction, which improved accuracy. Xi et al. [139] used an SDAE-BP model to diagnose combine harvester operational faults, achieving a diagnostic accuracy of up to 99%.

(3) Data Augmentation and Few-Shot Learning

**Figure 7:**
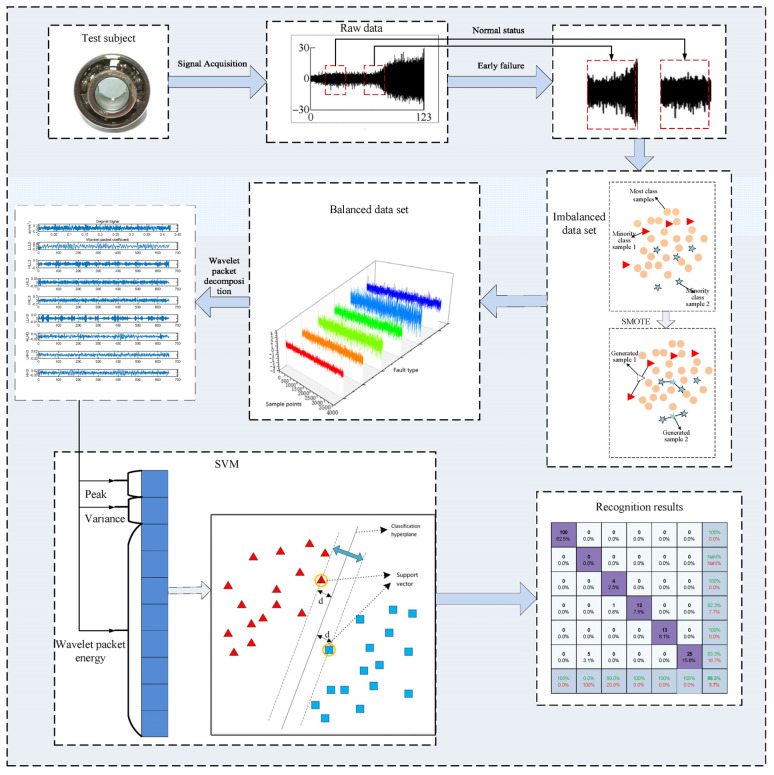
Early fault diagnosis method for rolling bearings using data augmentation [89].

To address the issue of limited fault samples in combine harvesters, data augmentation and specialized few-shot learning methods have been proposed. Xu et al. [140] used Time GAN for data augmentation to increase the number of imbalanced fault samples, which improved the performance of the diagnostic model. Xie et al. [89] employed the SMOTE technique to synthesize fault samples and balance the early fault dataset for bearings, effectively improving diagnostic accuracy and recall rate. The flowchart of their fault diagnosis method is shown in Figure 7. She et al. [9] proposed a meta-transfer learning driven few-shot fault diagnosis method aimed at diagnosing combine harvester gearbox faults with limited data, demonstrating the feasibility of the approach.

(4) Signal Fusion and Model Fusion

Combining data from different sensors or the outputs of different models can improve the comprehensiveness and accuracy of diagnosis. Data fusion can be performed at the signal level, feature level, or decision level. For example, Feijoo et al. [113] used a composite spectrum to fuse vibration signals from multiple accelerometers, effectively detecting rotational unbalance. Chen et al. [131] combined BPNN and DS Evidence Theory for blockage fault diagnosis, fusing neural network outputs and evidence reasoning, which improved diagnostic accuracy. Wang et al. [110], when diagnosing PMSM faults, constructed synchronous datasets of current and vibration signals using maximum cross-correlation and performed fusion at the feature level, improving diagnostic performance.

(5) Applications of Optimization Algorithms

Optimization algorithms are frequently employed to enhance diagnostic performance by improving model structures, parameters, or feature selection. Swarm intelligence algorithms such as Particle Swarm Optimization (PSO) [141], Genetic Algorithm (GA) [142,143], and Harmony Search (HS) have been used to optimize neural network weights [144] or for feature selection [145]. Bayesian optimization has been applied to hyperparameter tuning of deep learning models, leading to improved performance of diagnostic models [138].

Table 3 summarizes the main techniques, advantages, and disadvantages of data-driven methods applied in structural fault diagnosis of combine harvesters.

In summary, the evolution from classic models like SVM and NN to recent deep learning models such as CNNs and autoencoders reflects a trade-off between data requirements and feature engineering dependency. The success of classical models like SVM [81,130] lies in their effectiveness with small, high-dimensional datasets, making them a practical choice when fault data are extremely scarce. They are superior under these specific, data-limited conditions where complex deep learning models would overfit or fail to converge. The paradigm shift towards Deep Learning, particularly CNNs [135,137], is successful because these models automate the crucial, yet difficult, task of feature extraction. Their ability to learn hierarchical representations directly from raw or minimally processed data is a significant advantage when dealing with the complex, non-stationary signals from harvesters, for which handcrafted features are often unreliable. Thus, the superiority of deep learning is conditional on the availability of sufficient data and computational resources, while classical models remain a robust alternative for smaller-scale problems. Techniques such as data augmentation, few-shot learning, and multi-source signal/model fusion effectively mitigate the issues of data scarcity and limited single-source information. The application of optimization algorithms has also further enhanced model performance. However, the “black box” nature of complex models and their robustness under extreme working conditions remain topics requiring in-depth research.

### 3.4. Systems and Technologies Required for Implementing Data-Driven FDD

This subsection will discuss the practical system architectures and enabling technologies required to implement complex data-driven fault detection and diagnosis methods in combine harvesters and related scenarios. It will cover different deployment forms such as on-board diagnostic units, remote monitoring platforms, and automated testing systems, and explore key supporting technologies including hardware, software, communication, and simulation.

(1) On-Board Diagnostic Units and Real-Time Processing

Integrating data-driven FDD capabilities into on-board diagnostic units within the combine harvester is crucial for achieving real-time fault detection. These units typically consist of high-performance embedded hardware, data acquisition modules, and interfaces with the machine’s control system. Feng et al. [146] described a combine harvester on-board information platform with fault diagnosis capabilities, capable of acquiring operational data and performing diagnosis in real time. This system significantly improved the reliability of information acquisition for the driver, allowing for immediate alerts and thus reducing potential downtime and enabling proactive maintenance. Li et al. [147] developed an online monitoring system for combine harvester hydraulic actuators, integrating data acquisition, display, and alarming. This system was shown to monitor working parameters and alarm in real time, significantly improving machine operational efficiency by facilitating early fault detection. Li et al. [148] designed a fault diagnosis and load feedback control system for combine harvesters based on a microcontroller (SCM), utilizing speed ratio analysis for fault diagnosis. This work demonstrated the feasibility of on-board real-time FDD, directly contributing to increased machine availability during critical harvest periods. Implementing real-time signal processing and the efficient execution of diagnostic algorithms on resource-constrained on-board platforms is a significant challenge.

(2) Remote Monitoring and Management Platform

FDD platforms based on remote communication technology enable centralized monitoring and management of multiple combine harvesters. Such platforms typically consist of on-board units, communication networks, remote servers or cloud platforms, and user terminals. Bai et al. [24] designed and implemented a remote operation and maintenance platform for combine harvesters, which includes data monitoring, fault prediction, fault diagnosis, and integrated maintenance modules. They verified the real-time monitoring and diagnosis functions of the platform through field tests. De Almeida et al. [149] reported on a Telemetry System for Agricultural Machinery Management (T-SADA), which combines mobile communication (GSM/GPRS) and ZigBee radio for data transmission and provides fault tolerance. Qiu et al. [150] designed a remote monitoring system for combine harvesters based on multi-source information fusion, aiming to achieve effective monitoring, fault diagnosis, and remote scheduling. Sun et al. [121] proposed an agricultural machinery fault diagnosis scheme based on a remote distributed fault diagnosis system, including station monitoring, a remote diagnosis center, network communication, and software system development. Zhang et al. [151] developed a comprehensive operation and maintenance platform for combine harvesters, which achieved real-time processing of health status and fault diagnosis, improving the level of management. These platforms contribute to achieving fleet-level data analysis and maintenance management, enhancing the intelligent management level of combine harvesters.

(3) Application in Automated Testing Systems

Applying data-driven FDD methods to automated testing systems for combine harvester components or the entire machine can improve testing efficiency and diagnostic accuracy, ensuring product quality. These testing systems typically integrate various test instruments and software, capable of automatically executing test procedures and diagnosing faults. Sun et al. [152] designed and validated a combine harvester electrical system performance test bench, used for electrical system performance testing before leaving the production line, which improved detection efficiency. Zhang et al. [153] discussed Automated Test Systems (ATSs) for agricultural equipment built based on embedded computers and VXI/PXI modules, used for fault detection and isolation, which improved diagnostic capability and efficiency [154]. Zhang et al. [155] proposed a wireless rapid detection method for evaluating the vibration characteristics and mechanical structural defects of wheeled tractors under idle conditions, which can be used for pre-delivery quality inspection.

(4) The Role of Simulation and Modeling in the Implementation Process

Simulation and modeling play an important auxiliary role in the implementation process of data-driven FDD. They can help researchers understand complex fault mechanisms. For example, Feng et al. [15] studied the correlation between gear system wear and vibration. Simulation and modeling also help to validate proposed diagnostic methods. For example, Liu et al. [156] studied variable diameter threshing drums by combining mathematical models and simulation. Furthermore, simulation can also be used to optimize sensor layout or optimize designs to prevent faults. Abdeen et al. [157] used FEA to simulate the forces on the thresher top cover. Chen et al. [158] used CFD-DEM coupled simulation to study blockage during rice drying. These applications indicate that simulation and modeling are indispensable tools in the research and implementation process of data-driven FDD.

Implementing data-driven FDD requires constructing a system architecture comprising on-board diagnostic units and a remote monitoring platform, and relies on high-performance hardware, reliable communication, and advanced software and algorithms. Current research has explored various system design solutions and validated their effectiveness in laboratory and field tests. However, key issues that still need to be addressed in practical applications include reducing system cost and power consumption, improving system stability and usability, and achieving interoperability between different systems, all while ensuring real-time performance and accuracy. Future research should further focus on optimizing the computational efficiency of on-board units, enhancing the reliability of wireless communication, and developing techniques for seamlessly integrating FDD systems into existing combine harvester electrical architectures and information systems. The role of simulation and modeling as auxiliary tools in system design and validation should not be overlooked.

## 4. Current Major Challenges

Despite data-driven methods injecting new vitality and achieving significant advancements in the field of structural fault detection and diagnosis for combine harvesters, their practical application in complex, variable agricultural production environments still face numerous inherent and thorny challenges. These challenges stem from the unique characteristics of combine harvesters and the complexity of their operating environments, which significantly distinguishes them from fault diagnosis of industrial equipment in controlled environments [159,160,161,162,163,164,165]. They present significant challenges across multiple stages, including data acquisition, signal processing, model building, and practical deployment.

### 4.1. Distinctive Characteristics and Core Challenges in Combine Harvester FDD

(1) The Distinctive Characteristics of Combine Harvester

FDD for agricultural machinery, particularly combine harvesters, possesses distinctive characteristics that significantly differentiate it from other mechanical fields. Unlike factory equipment operating under relatively stable and controlled conditions, combine harvesters operate in highly unstructured and dynamic agricultural environments. These environments expose machinery to extreme dust, moisture, temperature fluctuations, severe and irregular vibrations, and continuous impacts. Furthermore, the operational loads and speeds of combine harvesters change drastically with varying terrain, crop types, and harvest conditions.

(2) Impact on Signal Patterns

These unique operational conditions profoundly influence the characteristics of signals acquired from combine harvesters. For instance, vibration signals are typically non-stationary, contain high background noise from engine and crop processing, and often exhibit complex, multi-source fault signatures that can be masked by normal operational impacts. Similarly, acoustic signals are heavily influenced by ambient noise and transient events, making fault pattern identification challenging. Force and strain signals also show highly dynamic and non-linear patterns, often making it difficult to distinguish fault-induced changes from normal load variations.

(3) Challenges for Signal Processing and Machine Learning Methods, etc.

The aforementioned signal characteristics present significant challenges for traditional FDD methodologies. Signal preprocessing methods must be robust enough to effectively denoise and de-trend non-stationary and noisy data without inadvertently removing subtle fault features. Consequently, feature extraction techniques need to be highly adaptive and capable of capturing transient and non-linear patterns across various domains (time, frequency, time–frequency), rather than relying on fixed or stationary features. For machine learning and artificial intelligence models, the primary challenge lies in developing models with superior robustness and generalization capability that can accurately diagnose faults under diverse, unseen operational conditions and with limited, imbalanced fault data.

(4) These complexities, coupled with the integrated nature of mechanical, hydraulic, electrical, and electronic systems within a combine harvester, pose higher demands on the protective capabilities of FDD systems. Ultimately, ensuring the robustness of signal processing methods and the adaptability and real-time performance of diagnostic models in such a challenging environment constitutes the primary hurdle for research and practical application in this field.

### 4.2. The Dilemma of Data Acquisition and Limitations in Data Quality Constitute a Fundamental Bottleneck

The dilemma of data acquisition and the limitation of data quality constitute a fundamental bottleneck for implementing data-driven FDD. Due to the relatively low frequency of structural faults in agricultural machinery, especially early and compound faults, high-quality data samples that comprehensively cover different fault types, severity levels, and working conditions are extremely scarce. This inherent scarcity and severe imbalance of data (where normal working condition data are far more abundant than fault data) directly limits the effective training of machine learning models that have a high reliance on large-scale data, particularly deep learning methods. Simultaneously, raw signals acquired in harsh environments are often contaminated by strong background noise, environmental interference, and multi-component coupling vibration. The signals exhibit a low signal-to-noise ratio (SNR) and show significant non-stationary and non-linear characteristics. How to ensure the authenticity and validity of the acquired data is a pressing issue.

### 4.3. Appropriate Sensor Selection and Robust Feature Extraction Constitute Core Technical Difficulties

Appropriate sensor selection and robust feature extraction represent core technical difficulties. For machines integrating numerous complex components like combine harvesters, the scientific selection of sensor types, quantities, and optimal installation positions to ensure the capture of signals strongly correlated with specific structural faults and possessing discriminative power is itself a complex system engineering task. A greater challenge lies in the fact that physical damage to the structure, such as initial fatigue cracks or slight wear, often exhibit complex, non-linear, and indirect relationships with the signals ultimately measured by the sensors. This relationship is readily masked by the complex dynamic loads generated during normal machine operation and environmental noise. This makes it exceedingly difficult to accurately extract physical quantities or abstract features that genuinely reflect the nature of the fault from the raw signals. The robustness and accuracy of existing signal processing techniques in handling the non-stationarity, non-linearity, and strong noise interference inherent in signals acquired from combine harvesters still require improvement. Effectively separating fault signals from normal fluctuations induced by changes in working conditions is a pressing issue that urgently needs to be addressed.

### 4.4. Insufficient Robustness and Generalization Capability of Diagnostic Models Limit Their Reliability in Practical Applications

The robustness and generalization capability of diagnostic models are still insufficient, which limits their reliability in practical applications. Diagnostic models trained in laboratory settings or on specific datasets often exhibit a dramatic decline in performance when confronted with the vastly changing loads, speeds, terrain, and crop conditions encountered during actual field operations, demonstrating poor generalization capability. These models show fragility towards factors such as unknown working conditions, environmental noise, and sensor drift, which can easily lead to high false positive or false negative rates, reducing user trust in the system. Furthermore, real-world faults often exist in compound forms, where multiple components simultaneously exhibit different types of damage and interact with each other. This makes the diagnosis of compound faults significantly more complex than that of isolated faults.

### 4.5. Practical Deployment and Large-Scale Application Face Realistic Barriers

Practical deployment and large-scale application of these technologies face realistic obstacles. Implementing real-time fault detection and diagnosis on resource-constrained on-board embedded platforms requires highly optimized algorithms and lightweight model structures. This presents a contradiction with some advanced data-driven models that have high computational complexity. Integrating FDD systems into existing combine harvester electronic control and information management systems also faces challenges related to technical interfaces, communication protocols, and system stability. The relatively high cost of high-performance sensors, data acquisition hardware, and computing platforms, coupled with the fact that advanced data-driven methods typically require a certain level of specialized background knowledge for effective application and maintenance, collectively constitute realistic barriers to the widespread adoption and independent use of this technology among a large number of farm users.

## 5. Future Research Directions

To address the aforementioned challenges and promote the further development of structural FDD technology for combine harvesters, future research should focus on the following directions.

(1) Construction and Sharing of High-Quality, Annotated Structural Fault Datasets Specific to Agricultural Machinery: Future efforts should prioritize establishing and promoting standardized datasets that encompass real-world operational data from diverse combine harvester models, covering various structural components, multiple fault types, and different severity levels under varied working and environmental conditions. Emphasis should be placed on accurate fault labeling and detailed metadata for each data point, which is critical for supervised learning. Furthermore, developing and integrating domain-specific digital twin models for critical combine harvester components with advanced simulation capabilities and generative models can create high-fidelity synthetic fault data to compensate for real-world data scarcity and enhance data diversity.
(2) Developing Robust and Adaptive Feature Extraction Methods for Combine Harvester Signals in Harsh Field Environments: In-depth research is needed on advanced signal processing techniques capable of effectively handling the unique characteristics of combine harvester signals, such as strong background noise, non-stationarity, and non-linearity. This includes exploring more advanced adaptive time–frequency analysis, robust modal decomposition techniques, and non-linear feature extraction methods that can effectively decouple subtle fault-related features from normal operational fluctuations and environmental interference. Research should focus on developing methods that can adaptively adjust to maintain feature robustness across drastically changing working conditions.
(3) Research on ML/AI Models Tailored for Combine Harvester Fault Scenarios: Future research should focus on developing ML/AI models specifically designed for rare fault events, data imbalance, and complex compound faults commonly encountered in combine harvesters (e.g., combined bearing wear and imbalance in the threshing unit). Advanced data augmentation and synthesis techniques should be developed to generate realistic fault samples. For compound faults, research should aim at developing diagnostic models and algorithms capable of effectively identifying and separating the individual fault sources and their complex causal relationships within the interconnected combine harvester systems.
(4) Enhancing the Explainability and Trustworthiness of Data-Driven Diagnostic Models for Combine Harvesters: Researchers should focus on developing explainable artificial intelligence (XAI) techniques applicable to combine harvester FDD to make the model’s diagnostic decision-making process more transparent and provide actionable insights to maintenance personnel and operators. Crucially, future research should focus on effectively incorporating agricultural engineering domain expert knowledge (e.g., machine design principles, crop mechanics, fault propagation paths specific to harvesters) into deep learning model structures or training processes to enhance the model’s physical interpretability and trustworthiness, thereby building greater user confidence.
(5) Integrated Multi-Source Heterogeneous Data and Model Information Fusion Techniques for Comprehensive Harvester Diagnostics: Researchers must develop advanced fusion algorithms and strategies capable of effectively integrating heterogeneous data from various sensors (e.g., vibration from threshing and vibrating screens, rotational speed from transmission, load/strain from header) and different data types. Explore multi-level fusion methods to enhance diagnostic accuracy, robustness, and comprehensiveness. Particular attention should be paid to the effective temporal synchronization and complementary utilization of information from multi-source data streams obtained under the challenging and complex working conditions of combine harvesters.

The data-driven FDD methods and proposed future directions are largely generalizable to other types of agricultural machinery (e.g., tractors, seeders, tillage equipment) that operate under similar challenging field conditions and exhibit complex structural interactions. The fundamental principles of data acquisition, signal processing, feature extraction, and ML/AI model application remain relevant. Therefore, insights gained from FDD research on combine harvesters can often be adapted and applied to enhance the reliability and efficiency of the broader agricultural machinery fleet.

## 6. Conclusions

This paper systematically reviewed the latest advancements in structural FDD for combine harvesters based on data-driven methods. The review first outlined the complex structure of combine harvesters and their common structural fault types, emphasizing the patterns of occurrence of these faults in harsh field environments and their critical impact on machine performance. Subsequently, it detailed the core steps of data-driven FDD methods, including data acquisition and preprocessing, signal processing and feature extraction, and the application of machine learning and artificial intelligence models. The exploration and application of these technologies provide important theoretical foundations and technical approaches for achieving intelligent maintenance, reliable operation, and enhanced safety for combine harvesters.

Despite the significant progress made by data-driven FDD methods in this field, numerous challenges remain in practical applications. Unlike controlled industrial settings, these machines operate in highly dynamic and harsh agricultural environments, resulting in unique signal characteristics: non-stationary, noisy, and multi-source coupled vibrations and loads. The harsh and variable field working conditions, the selection of appropriate sensors, the scarcity and imbalance of fault data, the complexity of acquired signals, the insufficient robustness of feature extraction, and the limitations in the generalization capability of diagnostic models all constitute technical difficulties that urgently need to be addressed. Furthermore, the cost of high-performance sensors and computing platforms, as well as practical deployment issues related to integrating FDD systems into existing combine harvester electronic architectures, should not be overlooked, as these factors limit the widespread adoption of this technology among a large number of farm users.

Looking ahead, studies on structural fault FDD for combine harvesters should focus on constructing high-quality, standardized, and open datasets, developing robust feature extraction methods capable of handling complex non-stationary signals and harsh environmental interference, exploring advanced machine learning models suitable for few-shot, imbalanced, and compound fault scenarios, and enhancing the explainability of diagnostic models and the reliability of their practical deployment. Through the integration of multi-source heterogeneous data and modal information fusion, and by deeply understanding the association between fault mechanisms and data features, it is expected to further enhance the accuracy and robustness of detection and diagnosis, ultimately providing strong support for achieving efficient and reliable operation and intelligent management of combine harvesters. These advanced FDD methods hold the key to significantly reducing costly operational downtime and improving agricultural productivity, thereby contributing to global food security and promoting agricultural sustainability.

## Figures and Tables

**Table 1:** The likelihood matrix of fault against harvester structure section (H—high, M—medium, L—Low).

Type of Fault	Header and Feeding Section	Threshing Section	Cleaning Section	Transmission System	Hydraulic System	Chassis Frame and Connecting Parts	Overall Likelihood of Individual Fault
Fatigue	M	H	M	H	L	H	H
Wear	H	H	M	H	M	M	H
Fracture	L	M	L	M	L	M	M
Blockage	H	M	H	L	L	L	M
Overall likelihood of fault	H	H	M	H	M	M	

**Table 2:** Summary and comparison of common signal types.

Signal Type	Advantages	Disadvantages	Suitability and Challenges in Agricultural Field Conditions
Vibration [99,100,101]	Rich information on rotating machinery (bearings, gears, imbalance); well-established diagnostic features	Sensor placement critical; susceptible to noise from machine operation and impacts; requires physical contact and robust mounting	Highly relevant for internal component health (e.g., threshing drum, gearbox). Challenges include sensor protection from dust/moisture/impact, and isolating fault signals from operational vibrations and variable loads/speeds
Acoustic [102]	Non-contact; can detect air/fluid leaks, some mechanical anomalies (e.g., knocking)	Highly susceptible to ambient noise; less specific than vibration; signal attenuation	Potentially useful for detecting loose parts or abnormal operational sounds. Major challenge is the extremely noisy agricultural environment (engine, mechanisms, crop interaction), requiring advanced noise cancelation and directional microphones
Strain/Force [103]	Direct indication of structural loads, stress concentrations, fatigue potential	Sensor placement critical; installation can be complex; susceptible to temperature variations if not compensated	Excellent for assessing structural integrity of frame, shafts, and high-load components. Challenges include sensor durability under continuous variable loads and harsh environments; complex calibration may be needed

**Table 3:** Summary and comparison of data-driven diagnostic methods.

Data-Driven Method	Key Techniques	Advantages	Disadvantages	Suitability and Challenges in Agricultural Field Conditions
Support Vector Machine [81,104,130]	Kernel function selection; applicable to small sample and high-dimensional data	Good performance on small sample and high-dimensional data; good generalization capability	Difficult to select optimal kernel function; low efficiency for large-scale training sets	Suitable when labeled fault data are scarce, provided robust features are extracted. Performance depends heavily on feature quality, which is a challenge with noisy agricultural data
Neural Networks [126,131,132,133,139]	Neural Network structure, dynamic adjustment of variable weights	Self-learning from samples; high diagnostic accuracy	Requires large amounts of historical data; poor generalization capability; insufficient robustness under complex working conditions	A good general-purpose classifier for agricultural FDD. Feature importance can guide understanding. Robustness to noisy features is an advantage
Data Augmentation [9,89,140]	Synthesis of fault samples; balancing imbalanced datasets; techniques based on generative models	Addresses the issue of fault sample scarcity; improves diagnostic model performance	Difficult to generate high-quality fault samples with sufficient diversity; augmented data may not perfectly reflect the complexity of real faults	Very promising when time–frequency representations of signals are used as input. Data scarcity is a major hurdle; data augmentation and transfer learning are crucial.
Signal Fusion [110,113,131]	Combining data from different sensors/models; signal, feature, and decision-level fusion; integration of heterogeneous data	Improves diagnostic comprehensiveness and accuracy; enhances robustness	Difficult to select effective fusion algorithms; integration of multi-source heterogeneous data (especially in harsh and complex environments) is complex	Useful for anomaly detection when specific fault labels are unavailable (common in agriculture). Denoising capabilities are beneficial. Requires careful threshold setting for anomaly detection

## Data Availability

The data and the related conclusions presented in this article were all derived from the Web of Science database and “CNKI” (China National Knowledge Infrastructure).
